# Acute Cardiotoxicity Induced by 5-Fluorouracil Successfully Treated With Levosimendan: A Case Report

**DOI:** 10.7759/cureus.89996

**Published:** 2025-08-13

**Authors:** Carlos Wu-Chin, Carlos Zúñiga-Orlich, Juliana Salas-Segura, Adrián Lostalo-González, Jeaustin Mora-Jiménez, Sebastián Arguedas-Chacón, Kevin Cruz-Mora, Esteban Zavaleta-Monestel

**Affiliations:** 1 Critical Care Medicine, Hospital Clínica Bíblica, San José, CRI; 2 Oncology, Hospital Clínica Bíblica, San José, CRI; 3 Cardiology, Hospital Clínica Bíblica, San José, CRI; 4 Research, Hospital Clínica Bíblica, San José, CRI; 5 Pharmacy, Hospital Clínica Bíblica, San José, CRI

**Keywords:** 5-fluorouracil (5-fu), acute heart failure, cardiotoxicity, chemotherapy, levosimendan

## Abstract

Cardiotoxicity is a rare but potentially life-threatening complication of 5-fluorouracil (5-FU), commonly presenting as chest pain or acute heart failure. We present the case of a 48-year-old woman with colon cancer who developed acute decompensated heart failure within 48 hours of completing her first cycle of FOLFOX chemotherapy (5-FU, oxaliplatin, and leucovorin). Echocardiography revealed a left ventricular ejection fraction (LVEF) of 28% and markedly elevated pro-B-type natriuretic peptide levels (>14,700 pg/mL), confirming severe cardiac dysfunction. The patient was admitted to the intensive care unit and received continuous levosimendan infusion at 0.1 µg/kg/minute for 24 hours, along with norepinephrine support. This treatment resulted in significant clinical improvement, normalization of cardiac biomarkers, as documented by serial laboratory tests, and full recovery of renal and cardiac function. Follow-up echocardiography showed improvement in LVEF to 50%. Other differential diagnoses, such as ischemic heart disease, sepsis, and myocarditis, were ruled out through clinical, laboratory, and imaging findings. Although causality cannot be definitively established, this case highlights the potential utility of levosimendan in chemotherapy-related cardiotoxicity, particularly in patients requiring inotropic support. Only limited case-based literature exists regarding its use in this context. The patient remained clinically stable and asymptomatic during four weeks of outpatient follow-up. Further clinical studies are warranted to establish evidence-based recommendations.

## Introduction

Fluoropyrimidines (FPs), specifically capecitabine and 5-fluorouracil (5-FU), are drugs commonly used to treat solid tumors, especially those of the gastrointestinal tract. These medications were first discovered in 1957 by Heidelberger et al., who induced tumor cell death using 5-FU for the first time. Although approved in 1962 for the treatment of colon, rectal, breast, stomach, and pancreatic adenocarcinomas, their use became widespread over time [1,2].

Among the most common adverse effects of FPs are myelosuppression, gastrointestinal disturbances, and hand-foot syndrome, which began to be documented following their widespread use in clinical practice. One of the less frequent but clinically significant adverse events is cardiotoxicity, which typically presents as unstable angina. The incidence of cardiovascular events associated with FPs varies widely, ranging from 0% to 34.6%, while more severe manifestations, such as cardiomyopathy, myocarditis, and sudden cardiac death, occur in approximately 0% to 2% of cases. Currently, the definition of cardiotoxicity is not limited to clinical symptoms but also includes a reduction in left ventricular ejection fraction (LVEF) and histopathological changes in cardiomyocytes [1,2].

Levosimendan is a calcium sensitizer that provides short-term inotropic support for patients with acute decompensated heart failure who cannot be treated conventionally. Its positive inotropic effect does not increase myocardial oxygen consumption, which improves coronary blood flow while reducing both preload and afterload. This mechanism provides a cardioprotective effect, shielding the heart from ischemia-reperfusion injury [3].

To date, there is no substantial evidence on the use of levosimendan in 5-FU-induced cardiomyopathy, and case reports are limited. This article aims to describe the case of a patient who developed heart failure after FOLFOX chemotherapy (5-FU, oxaliplatin, and leucovorin) and was successfully treated with levosimendan when low cardiac output led to cardiovascular collapse, with a decreased LVEF.

## Case presentation

A 48-year-old female patient, weighing 56 kg, with no relevant personal medical history, was admitted due to acute decompensated heart failure following the first cycle of FOLFOX chemotherapy as part of her treatment for colon carcinoma. During the sigmoidectomy, significant lymphatic dissemination was identified, with 5 out of 23 lymph nodes testing positive, although complete resection of both the primary tumor and the affected lymph nodes was achieved.

After surgery, she was administered the FOLFOX regimen as adjuvant chemotherapy and was managed on an outpatient basis for 48 hours at home. However, since the start of the treatment, the patient developed symptoms of chest pain, anorexia, and vomiting, leading to acute kidney injury, with an increase in creatinine to 2.0 mg/dL. This prompted the patient’s hospitalization for proper management.

**Figure 1:**
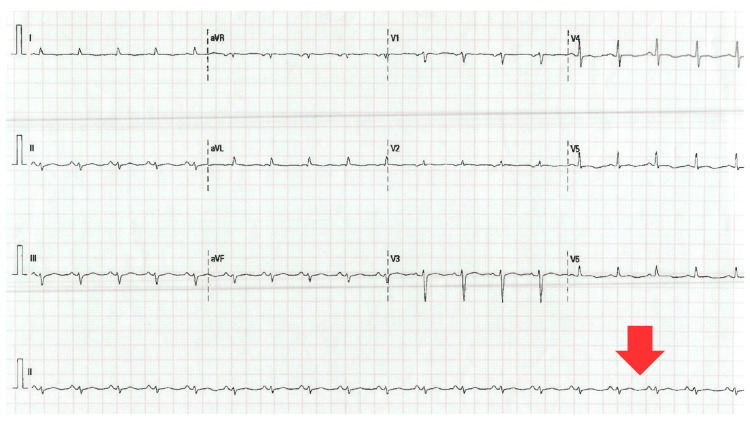
Electrocardiogram showing low voltage in limb leads in the context of 5-fluorouracil-induced cardiotoxicity.

On June 25, she was urgently admitted due to the worsening of her symptoms, presenting with tachypnea and a tendency to desaturate, which required the initiation of high-flow oxygen therapy to maintain adequate oxygenation. On June 26, an echocardiogram was performed, revealing significant depression of ventricular function, with a drop in LVEF to 28%. The electrocardiogram (Figure 1) showed low-voltage QRS complexes in the peripheral leads, a finding consistent with diffuse myocardial involvement.

**Figure 2:**
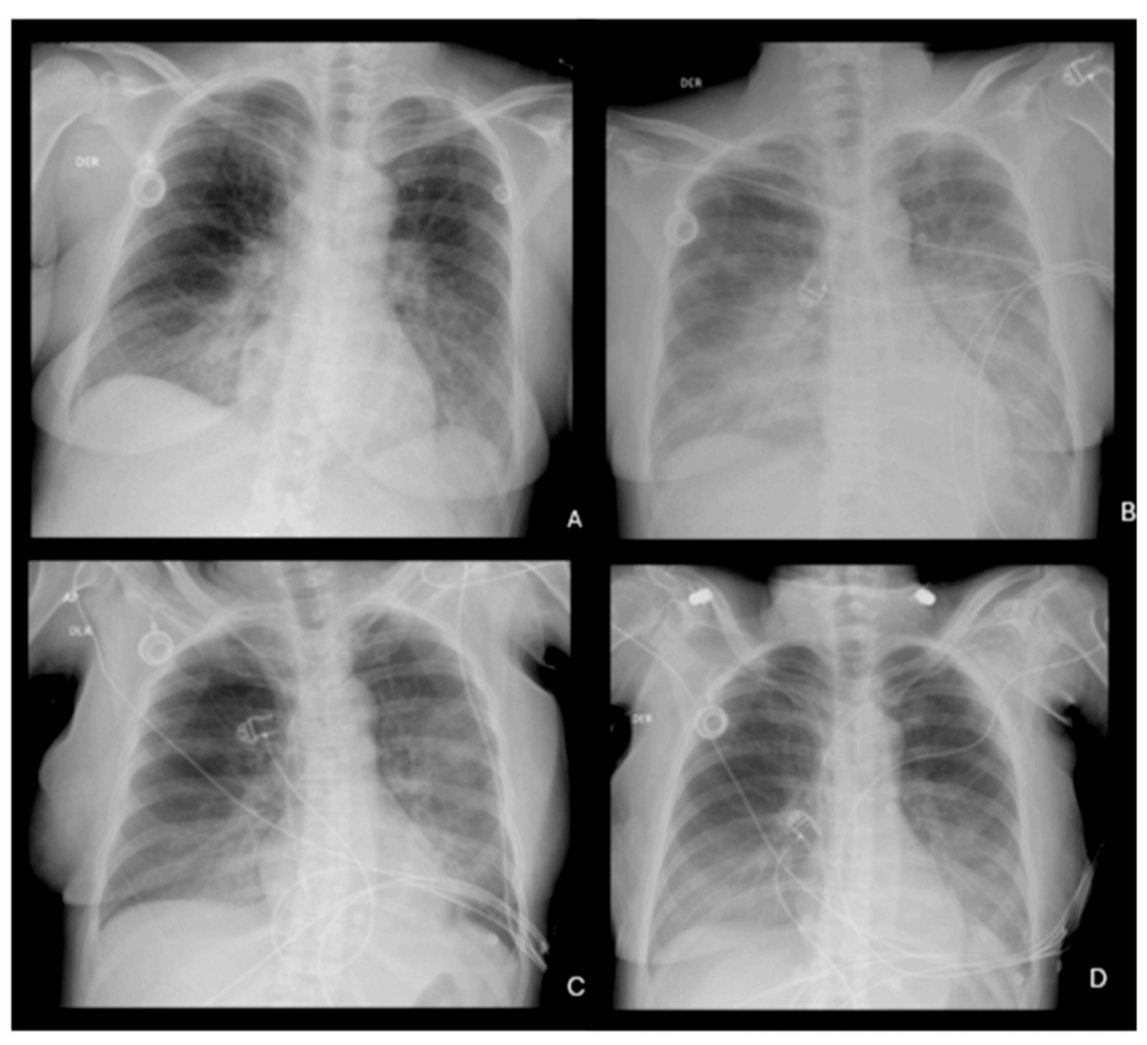
Chest X-ray shows bilateral pulmonary congestion with progressive worsening compared to the previous day. (A) Chest X-ray at admission before transfer to the intensive care unit (ICU) (day one). (B) ICU admission with signs of bilateral pulmonary congestion (day two). (C) Radiological improvement after starting levosimendan (day three). (D) Progressive resolution of congestion with 12 mL/hour of levosimendan and support with norepinephrine (day four).

Additionally, the chest X-ray showed bilateral pulmonary congestion with progressive worsening compared to the previous day (Figures 2A, 2B). This finding, along with elevated pro-B-type natriuretic peptide (pro-BNP) levels greater than 14,700 pg/mL, was consistent with acute heart failure, possibly related to chemotherapy-induced cardiotoxicity, particularly from 5-FU.

Given the severity of the situation, the patient was transferred to the intensive care unit (ICU), where non-invasive mechanical ventilation was initiated, and a four-lumen central catheter was placed via the left subclavian vein under aseptic conditions and local anesthesia to facilitate the administration of the necessary medications. Additionally, treatment with levosimendan was started to improve ventricular function and counteract the 5-FU-induced cardiotoxicity. This was initially administered as an intravenous bolus, followed by a continuous infusion titrated from 3 to 12 mL/hour.

On June 27, an improvement in the radiological signs of bilateral pulmonary congestion was observed (Figure 2C). However, a deterioration in renal function was noted, with nitrogen retention and creatinine levels exceeding 4 mg/dL, as well as hyperphosphatemia (Table 1). Despite maintaining normal blood pressure with norepinephrine at 5 mL/hour, the infusion rate of levosimendan was increased from 3 to 5 mL/hour. That same night, the therapeutic goal of administering levosimendan at 12 mL/hour was achieved without triggering hypotension, owing to the concomitant administration of norepinephrine as a preventive measure. This intervention resulted in a 30% ejection fraction.

**Table 1:** Trends in laboratory parameters during hospitalization. Na^+^: sodium; K^+^: potassium; Cl^⁻^: chloride; Cr: creatinine; BUN: blood urea nitrogen; AST: aspartate aminotransferase; ALT: alanine aminotransferase; Pro-BNP: pro-B-type natriuretic peptide; NR: not reported (laboratory parameters were not ordered for these dates)

Parameter	07/25/25	07/26/25	07/27/25	07/28/25	07/29/25	07/30/25	07/01/25	Reference value
Na^+^ (mmol/L)	130.7	129.8	131.7	133.5	138.6	138.8	139.3	136–145
K^+^ (mmol/L)	4.6	4.38	4.51	3.58	4.16	4.9	4.9	3.5–5.1
Cl^⁻^ (mmol/L)	92	98.4	94	NR	NR	NR	106.8	98–107
Cr (mg/dL)	2.07	3.56	4.24	2.96	1.71	1.21	0.988	06–1.3
BUN (mg/dL)	35.3	47.7	88.9	76	47.8	28.8	24.6	6–20
Phosphorus (mg/dL)	6.85	6.85	6.82	4.57	2.68	2.1	3.18	2.5–4.5
AST (U/L)	57.9	57.9	57.9	34	NR	NR	NR	<35
ALT (U/L)	55.2	47.8	47.8	48.3	NR	NR	NR	<35
Total bilirubin (mg/dL)	0.589	0.513	0.513	0.6	NR	NR	NR	<1.2
Pro-BNP (pg/mL)	NR	14,753	14,202	7,310	4,500	3,405	1,802	<300

On June 28, the patient showed satisfactory progress, with no sinus tachycardia, maintaining a heart rate of less than 100 beats/minute, normotensive, without vasopressor support, and receiving a maximum dose of 12 mL/hour of levosimendan based on her weight, with a mean arterial pressure of 83 mmHg. A significant decrease in pro-BNP was achieved, dropping to half of the initial recorded levels (Table 1). The chest X-ray showed reduced opacity in both lung fields (Figure 2D), along with an improvement in renal function, with creatinine decreasing to 2.9 mg/dL.

**Figure 3:**
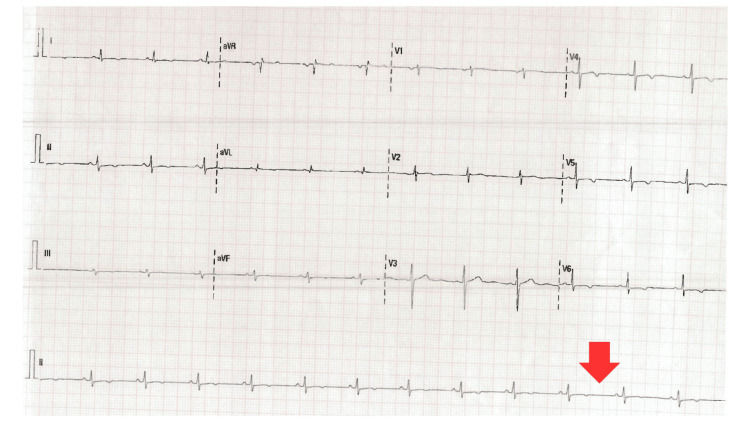
Electrocardiogram showing normalization of QRS voltage following the clinical resolution of 5-fluorouracil-induced heart failure.

After her stay in the ICU, the patient was transferred to intermediate care, where she showed progressive improvement in renal function over the following days, with a decrease in creatinine levels to 0.988 mg/dL. A significant reduction in pro-BNP levels was also observed, reaching 1,802 pg/mL on her last day of hospitalization. Additionally, a normal electrocardiogram was recorded (Figure 3), and a Doppler echocardiogram revealed an LVEF of 50%. The patient was subsequently successfully discharged, with complete resolution of both acute kidney injury and acute decompensated heart failure.

## Discussion

Mechanism of 5-FU toxicity

The cardiotoxicity related to 5-FU is primarily attributed to coronary vasospasm, a theory suggesting that 5-FU chemotherapy may cause spasm in the coronary arteries, leading to myocardial ischemia. This vasospasm, which can be detected via electrocardiogram (ST-segment elevation) and elevated troponins without evidence of macrovascular coronary disease in angiography, may be caused by endothelial dysfunction (endothelium-dependent) or smooth muscle dysfunction (endothelium-independent) [4,5].

Endothelial dysfunction occurs when the damaged endothelium is unable to release nitric oxide, which normally induces vasodilation, leading to paradoxical vasoconstriction. On the other hand, smooth muscle dysfunction can also cause vasoconstriction, even with a functionally intact endothelium. Although coronary vasospasm related to 5-FU is a plausible theory, it is not always consistently observed in angiographic studies. However, some studies have shown vasoconstriction in peripheral arteries, which could correlate with coronary vasospasm [6,7].

Other proposed mechanisms include direct damage to cardiomyocytes due to the generation of reactive oxygen species and oxidative stress, which induce apoptosis of myocardial cells, as well as alterations in iron homeostasis and the activation of ferroptosis, a type of iron-dependent cell death associated with lipid peroxidation [8,9].

Mechanism of action of levosimendan in reversing cardiotoxicity

**Figure 4:**
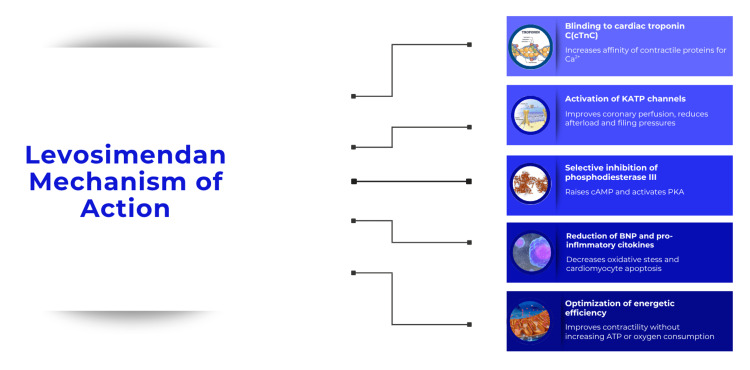
Mechanisms of action of levosimendan. Authors’ own image.

Levosimendan exerts a multifaceted cardioprotective effect that extends well beyond its classical inotropic action. Its primary mechanism of action, illustrated in Figure 4, involves calcium sensitization via troponin C in a calcium-dependent manner. This enhances myocardial contractility without increasing intracellular calcium concentration or energy consumption, thereby reducing the risk of arrhythmias and myocardial stress [10,11].

In addition, levosimendan activates ATP-sensitive potassium channels in mitochondria, triggering effects similar to ischemic preconditioning, which help limit ischemia-reperfusion injury and promote functional recovery. The drug also exhibits anti-inflammatory, antioxidant, and anti-apoptotic properties by reducing the expression of pro-inflammatory cytokines such as interleukin 6 and tumor necrosis factor-alpha, as well as mitigating oxidative stress and cardiomyocyte apoptosis [10,11].

Levosimendan improves diastolic relaxation (positive lusitropic effect), optimizing ventricular filling without compromising diastolic function. It also favors ventriculo-arterial coupling by increasing contractility and reducing afterload, thereby enhancing cardiovascular efficiency. In experimental models, it has been shown to prevent cardiac remodeling by inhibiting hypertrophy, apoptosis, and changes in gene expression related to myocardial aging. Finally, it contributes to better coronary perfusion through nitric oxide-mediated vasodilation and protects against vasospasm induced by vasoconstrictive agents [12,13].

In this case, levosimendan was considered due to its cardioprotective effects, which are not observed with other inotropes such as milrinone or dobutamine. Regarding the safety profile, levosimendan and milrinone share similar characteristics, with hypotension being one of the most common adverse effects. However, this can be effectively managed through careful dose titration [14].

Literature review and future perspectives

Current scientific evidence on the use of levosimendan for the prevention or treatment of chemotherapy-induced cardiotoxicity remains limited. One of the earliest reports available was published by García et al. in 2006, documenting the case of a 42-year-old patient with acute decompensated cardiomyopathy secondary to doxorubicin in the setting of metastatic breast cancer. Administration of levosimendan led to a marked clinical improvement observed just two days after the infusion began, setting a precedent for exploring this drug within the emerging field of cardio-oncology [15].

Since then, additional clinical experiences have been reported that further support the potential therapeutic benefit of levosimendan in this context. For instance, the case of a 49-year-old woman with an LVEF of 15% has been described, in which three cycles of levosimendan were administered during a 70-day hospital stay, resulting in progressive recovery. Although anecdotal, these reports suggest a potential role of levosimendan in patients with severe ventricular dysfunction induced by anthracyclines [3].

Regarding other FPs, such as capecitabine, its frequent combination with oxaliplatin, a drug with a relatively low cardiotoxic potential, has been documented. Most studies do not report significant cardiac events in patients without pre-existing cardiovascular disease. However, acute cardiac events have been observed, particularly when these agents are administered concurrently, underscoring the need for close clinical monitoring in high-risk patients [16,17].

To date, no evidence in the literature supports the use of levosimendan for the reversal or prevention of oxaliplatin-induced cardiotoxicity specifically. Existing studies have focused primarily on doxorubicin-related myocardial injury, a chemotherapeutic agent with a well-established cardiotoxic profile [14].

Despite these encouraging findings, the lack of controlled clinical trials in this indication limits the ability to draw definitive conclusions. Well-designed randomized controlled trials and long-term follow-up studies are essential to clearly define the safety profile, therapeutic efficacy, and specific indications of levosimendan in the setting of cardiotoxicity induced by FPs, anthracyclines, or other chemotherapeutic agents.

## Conclusions

This case illustrates a favorable clinical course following the administration of levosimendan in a patient with presumed 5-FU-induced cardiotoxicity. Although a definitive causal relationship cannot be established, the observed improvement supports the potential therapeutic role of levosimendan in patients with chemotherapy-induced ventricular dysfunction, particularly those requiring inotropic support. The conclusion primarily applies to 5-FU-related cardiotoxicity, though similar mechanisms may extend to agents such as capecitabine and anthracyclines. This report is limited by its single-patient design and lack of mechanistic confirmation. Future research should include prospective observational studies and randomized controlled trials to evaluate the efficacy and safety of levosimendan in this context. Potential clinical scenarios for consideration in future guidelines include patients with acute decompensated heart failure after chemotherapy, reduced ejection fraction unresponsive to standard therapy, or suspected myocardial stunning without ischemia.
